# Synthesis of New Racemic α,α-Diaminocarboxylic Ester Derivatives

**DOI:** 10.3390/molecules15129354

**Published:** 2010-11-17

**Authors:** Mabrouk El Houssine, Elachqar Abdelrhani, Alami Anouar, El Hallaoui Abdelilah

**Affiliations:** Laboratoire de Chimie Organique Fès, Faculté des Sciences Dhar El Mahraz, Université Sidi Mohamed Ben Abdellah, Morocco

**Keywords:** amine, *N*-alkylation, methyl α-azidoglycinate, α-amino acids

## Abstract

New racemic methyl or ethyl α-aminoglycinate derivatives were synthesized by *N*-alkylation of amines (aniline, 4-methylaniline, 2-methylaniline, 2,4-dimethoxyaniline, 2-nitroaniline, 4-chloro-2-fluoroaniline, 2-naphthylamine, benzylamine, *N*,*N*-dibenzylamine, and cyclohexylamine) with methyl or ethyl α-azidoglycinate.

## 1. Introduction

Amino acids are the fundamental building blocks of peptides and proteins and play essential roles in living organisms. Because of the physiological importance of α-amino acids, innumerable studies for their chemistry and synthesis have been published [1,2,3,4]. Along with the elucidation of their distributions, origins and physiological functions, the D-amino acids have been recognized as the candidates for novel physiologically active substances and/or marker molecules of diseases [5,6].

Since the end of last century, the studies of amino acids have changed to focusing on their biochemistry, physiology and medical activities such as apoptosis inducing, platelet aggregation-inhibiting/inducing, antimicrobial, anti-HIV.

The synthesis of amino acids was a very important development [7]. Their applications can be found currently in several domains: biochemistry, enzymology [8,9,10,11], medicine (antibiotics, antiepileptics, antivirals, antiprotozoals, cardiovascular, atherosclerosis, renal failure and diabetes, neuroexciters [12,13,14,15,16,17,18,19,20,21,22,23,24,25,26]), agrochemical industry (herbicides, fungicides, regulation of plant growth), in addition to their important utility as chiral auxiliaries in asymmetric synthesis [27,28]. The development of simple, efficient and highly selective methods for widely used organic compounds from readily available reagents is one of the major challenges in organic synthesis. Among these, C-N bond formation is one of the most important transformations. The reactions of amines have been a topic of immense research interest due to their synthetic utility [29,30,31,32,33] and biological activity [34]. Amines are widely used as intermediates to prepare solvents, fine chemicals, agrochemicals, pharmaceuticals and as polymerization catalysts [35,36,37].

Diaminoacids are important non-protein amino acids, usually components of both natural and synthetic bioactive compounds [38]. In fact, they are currently well recognized as key structural moieties in a variety of biologically active molecules [39,40].

The literature [41,42,43,44,45,46,47,48,49] reports different methods of alkylation, among which phase transfer catalysis, the *N*-alkylation by microwaves, and the Mitsunobu reaction with organic azides have proved to be efficient key intermediates in organic synthesis for the construction of heterocyclic systems by cycloaddition reactions, while the substitution of the azide group has received much less attention. Continuing our investigations on the use of organic azides [50,51], we report in this paper another part of our investigations concerning the preparation of new α,α-diaminocarboxylic esters derivatives with the aim of providing access to new active biomolecules. 

## 2. Results and Discussion

**Scheme 1:**
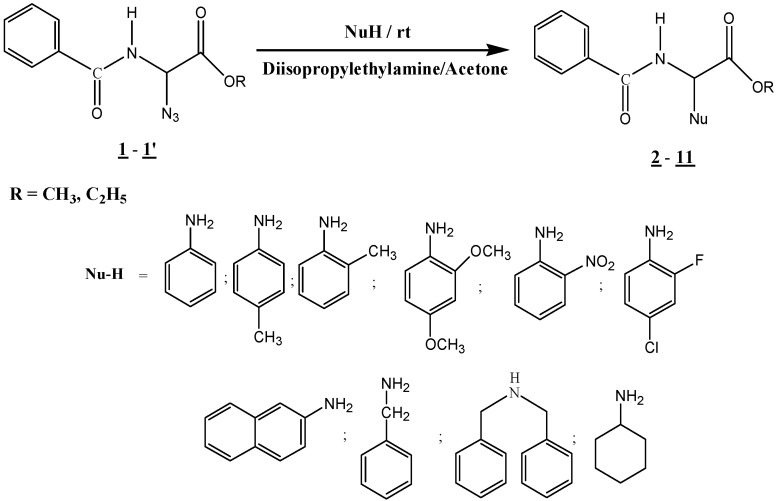
*N*-alkylation of different amines, with methyl or ethyl α-azidoglycinate **1** (R = CH_3_) or **1**’ (R = C_2_H_5_).

Our strategy is based on the *N*-alkylation of amines with methyl or ethyl α-azidoglycinate **1** (R = CH_3_) or **1**’ (R = C_2_H_5_) (Scheme 1). Azide derivatives **1** and **1**’ were prepared using Achamlale’s version of the Steglich reaction [52] and [53,54]. The title compounds are stable and can be stored for an unlimited amount of time without any signs of decomposition. Methyl or ethyl α-bromoglycinate also can be used and give satisfactory results; the azide **1** (R = CH_3_) or **1**’ (R = C_2_H_5_) are used especially for their stability.

As shown in Scheme 1, the *N*-alkylation reactions of various amine nucleophiles (aniline, 4-methylaniline, 2-methylaniline, 2,4-dimethoxyaniline, 2-nitroaniline, 4-chloro-2-fluoroaniline, 2-naphthylamine, benzylamine, *N*,*N*-dibenzylamine, and cyclohexylamine) with *N*-benzoylated methyl or ethyl α-azido-glycinates **1** (R = CH_3_) or **1**’ (R = C_2_H_5_) were performed in dry acetone for 48 h at room temperature in the presence of diisopropylethylamine (DIPEA). The products **2-11** were obtained in good to high chemical yields (62-92%) and were characterized by MS, ^1^H-NMR and ^13^C-NMR spectroscopy. The results are summarized in Table 1.

**Table 1:** Synthesis of new methyl or ethyl α-aminoglycinates derivatives **2-11**.

Entry	Nu-H	Product	m.p. (°C)	Reaction Time (h)	Yield (%)	δ_Hα_ (ppm)
1	Aniline	Methyl 2-benzamido-2-(phenylamino)acetate (**2**)	124-126	48	80	6.22
2	4-methylaniline (R = Me)	Methyl 2-benzamido-2-(*p*-tolylamino)acetate (**3**)	140-142	48	90	6.14
3	4-methylaniline (R = Et)	Ethyl 2-benzamido-2-(*p*-tolylamino)acetate (**3**’)	164-166	48	80	6.11
4	2-methylaniline	Methyl 2-benzamido-2-(*o*-tolylamino)acetate (**4**)	128-130	48	90	6.22
5	2,4-dimethoxy-aniline	Methyl 2-benzamido-2-(2,4-dimethoxyphenylamino)acetate (**5**)	170-172	48	86.5	6.15
6	4-chloro-2-fluoro-aniline	Methyl 2-benzamido-2-(4-chloro-2-fluorophenylamino)acetate (**6**)	152-154	48	90	6.20
7	2-nitroaniline	Ethyl 2-benzamido-2-(2-nitrophenylamino)acetate (**7**)	173-174	48	86	6.82
8	2-naphthylamine	Ethyl 2-benzamido-2-(2-naphthylamino)acetate (**8**)	210-212	48	62	6.25
9	Benzylamine	Methyl 2-benzamido-2-(benzylamino)acetate (**9**)	104-106	48	76	5.56
10	*N*,*N*-dibenzyl-amine	Methyl 2-benzamido-2-(*N,N*-dibenzylamino)acetate (**10**)	130-132	48	84	5.58
11	2-amino-cyclohexane	Methyl 2-benzamido-2-(cyclohexylamino)acetate (**11**)	182-184	48	92	5.72

Comparing these results with only the work done by our team [41,50,51], we see that we obtained almost identical results. The molecule **2** used for previous work as a reference model. In continuation of that work, we studied herein the influence of donors and withdrawing groups on the the aromatic ring of the aniline moiety on the NMR chemical shifts of these systems. In molecule **2** used as a reference, the proton on the carbon α to the carboxylate group appears as a doublet at 6.22 ppm. When the aromatic ring bears a donor group in positions 2 or 4 (entries 2, 3 and 4), the chemical shift of the α-proton does not undergo any major changes (δ_CH_ = 6.14-6.20 ppm).

It should be noted that in the ^1^H-NMR spectrum of pure product **6** the α-proton at 6.20 ppm appears as a multiplet. The chemical shift is practically the same as that of the α-proton in the reference molecule **2**, and the splitting is probably due to the opposite effects (+ M) and (− I) of the two halogen atoms (fluorine in position 2 and chlorine in position 4) on the aromatic ring.

In molecule **7**, the α-proton multiplet underwent a deshielding of 6.22 to 6.82 ppm due to the presence of an electron withdrawing group [with inductive and mesomeric effects (− I, − M)] at the *ortho* position of the aromatic aniline system. In the case of benzylamine, dibenzylamine and cyclohexylamine as *N*-alkylation reagents, the chemical shift of the α-hydrogen undergoes a shielding of about 0.60 ppm (6.22 to 5.62 ppm) compared to molecule 2 used as a reference model (products **9**, **10** and **11**). This can be justified by the electrondonor effect (+ I) of the benzyl and cyclohexyl groups.

In conclusion, this method provides a convenient method and easy procedure for the preparation of new methyl or ethyl α-aminoglycinate derivatives starting from the appropriate azide derivatives **1** (R = CH_3_) or **1**’ (R = C_2_H_5_). The *N*-alkylation of various amines (aniline, 4-methylaniline, 2-methylaniline, 2,4-dimethoxyaniline, 2-nitroaniline, 4-chloro-2-fluoroaniline, 2-naphthylamine, benzylamine, *N*,*N*-dibenzylamine, and cyclohexylamine) with azide occurred under very mild conditions and led after a reaction time of about 48 h to the desired products in very satisfactory yields (Table 1).The nucleophilic nature of the reagents has an immediate influence on the α-hydrogen acidity and its possible effect the reactivity of the α,α-diamino acids.

## 3. Experimental

### 3.1. General

Melting points were determined with an Electrothermal melting point apparatus and are uncorrected. NMR spectra (^1^H, ^13^C) were recorded on a Bruker AM 300 (operating at 300.13 MHz for ^1^H, at 75.47 MHz for ^13^C) spectrometer (Centre Universitaire Régional d’Interface, Fès). NMR data are listed in ppm and are reported relative to tetramethylsilane (^1^H, ^13^C); residual solvent peaks being used as internal standard. All reactions were followed by TLC. TLC analyses were carried out on 0.25 mm thick precoated silica gel plates (Merck Fertigplatten Kieselgel 60F254) and spots were visualised under UV light or by exposure to vaporised iodine. Mass spectra were recorded by electrospray on a micromass ESl Platform II (Université Montpellier II, France) and on a PolarisQ Ion Trap GC/MSn Mass Spectrometer (Centre Universitaire Régional d’Interface, Fès). Methyl α-azidoglycinate **1** (R = CH_3_) or **1**’ (R = C_2_H_5_) was prepared using Achamlale’s method [53,54].

### 3.2. Typical Procedure for N-Alkylation

To a stirred solution of amine (2.86 mmol) and diisopropylethylamine (3.12 mmol) in dry acetone (10 mL), α-azidoglycinate (2.6 mmol) was added. The mixture was stirred at room temperature and the reaction was followed by TLC (Kiesegel Merck 60F524). The solvent was evaporated under reduced pressure. The residue was quenched with saturated aqueous solution of ammonium chloride (20 mL) and extracted with dichloromethane(20 mL × 3). The organic phase was dried in sodium sulfate (Na_2_SO_4_) and the solvent was removed under reduced pressure. The product was purified by column chromatography on silica gel using ether/hexane as eluant to afford pure *N*-alkylated product. 

*Methyl 2-benzamido-2-(phenylamino)acetate* (**2**): Yield 80%; Solid, m.p. 124-126 °C (ether/hexane); R_f_ = 0.75 (ether); ^1^H-NMR (CDCl_3_, ppm): δ = 7.80 (m, 2H, H_arom_), 7.56-7.49 (m, 4H, H_arom_), 7.15-7.09 (m, 2H, NH_amid_ + H_arom_), 6.79-6.68 (m, 3H, H_arom_), 6.22 (d, 1H, H_α_, 8 Hz), 4.91 (br s, 1H, NH), 3.87 (s, 3H, OCH_3_); ^13^C-NMR (CDCl_3_, ppm): δ = 170.0, 166.7 (CO), 146.2, 133.7, 132.2 (2C), 129.4, 128.8 (2C), 127.9 (2C), 118.0, 113.5 (2C) (C_6_H_5_ aromatic carbons), 60.9(-CH-), 53.0 (CH_3_O); MS (electrospray) *m/z*: 285.2 (M + 1, 9%), 164 (100%), 104 (21%); (Formula: C_16_H_16_N_2_O_3_).

*Methyl 2-benzamido-2-(p-tolylamino)acetate* (**3**): Yield 90%; Solid, m.p. 140-142 °C (ether/hexane); R_f_ = 0.67 (ether); ^1^H-NMR (CDCl_3_, ppm): δ = 7.76 (m, 2H, H_arom_), 7.52-7.40 (m, 3H, H_arom_), 7.02 (m, 2H, NH_amid_ + H_arom_), 6.77-6.67 (m, 3H, H_arom_), 6.14 (d, 1H, H_α_, 7.8 Hz), 4.90 (br s, 1H, NH), 3.86 (s, 3H, OCH_3_), 2.24 (s, 3H, CH_3_); ^13^C-NMR (CDCl_3_, ppm): δ = 170.5, 167.2 (CO), 141.6, 133.2, 132.1, 130.0 (2C), 129.0, 128.7 (2C), 127.2 (2C), 114.2 (2C) (C_6_H_5_ aromatic carbons), 61.1 (-CH-), 53.4 (OCH_3_), 20.5 (CH_3_); EIMS *m/z*: 298 (M^+.^, 12%), 239 (88%), 122 (38%), 105 (100%), 77 (59%); (Formula: C_17_H_18_N_2_O_3_).

*Ethyl 2-benzamido-2-(p-tolylamino)acetate* (**3’**): Yield 80%; Solid, m.p. 164-166 °C (ether/hexane);R_f_ = 0.72 (ether); ^1^H-NMR (CDCl_3_, ppm): δ = 7.75 (m, 2H, NH_amid_ + H_arom_), 7.52-7.40 (m, 3H, H_arom_), 7.02 (m, 2H, NH_amid_ + H_arom_), 6.82-6.69 (m, 3H, H_arom_), 6.11 (d, 1H, Hα, 7.9Hz), 4.90 (br s, 1H, NH), 4.32 (q, 2H, OCH_2_, 7.05Hz), 2.25 (s, 3H, CH_3_), 1.33 (t, 3H, CH_3_, 7.05Hz); ^13^C-NMR (CDCl_3_, ppm):δ = 170.0, 167.3 (CO), 141.7, 133.4, 132.0, 130.0 (2C), 128.9, 128.6 (2C), 127.1 (2C), 114.3 (2C) (C_6_H_5_ aromatic carbons), 62.6 (-CH-), 61.3 (CH_3_CH_2_O), 20.5 (CH_3_), 14.9 (CH_3_CH_2_O); MS (electrospray) *m/z*: 313.2 (M + 1, 11.81 %); 239.1 (88.58%), 192.1 (11.82%), 136.1(37.80%), 118.0 (59.06%), 105.0 (100%); (Formula: C_18_H_20_N_2_O_3_).

*Methyl 2-benzamido-2-(o-tolylamino)acetate* (**4**): Yield 90%; Solid, m.p. 128-130 °C (ether/hexane); R_f_ = 0.62 (ether);^1^H-NMR (CDCl_3_, ppm): δ = 7.85 (m, 2H, H_arom_), 7.56-7.41 (m, 3H, H_arom_), 7.15-7.09 (m, 2H, NH_amid_ + H_arom_), 6.79-6.68 (m, 3H, H_arom_), 6.22 (d, 1H, H_α_, 8 Hz), 5.05 (br s, 1H, NH), 3.88 (s, 3H, OCH_3_), 2.24 (s, 3H, CH_3_); ^13^C-NMR (CDCl_3_, ppm): δ = 170.6, 167.2 (CO), 142.2, 133.2, 132.2, 130.6, 128.7 (2C), 127.4, 127.2 (2C), 123.1, 119.2, 111.3 (C_6_H_5_ aromatic carbons), 60.7 (-CH-), 53.4 (OCH_3_), 17.5 (CH_3_); EIMS *m/z*: 298 (M^+.^,14%), 239 (87%), 122 (64%), 105 (100%), 77 (69%); (Formula: C_17_H_18_N_2_O_3_).

*Methyl 2-benzamido-2-(2,4-dimethoxyphenylamino)acetate* (**5**): Yield 86.5%; Solid, m.p. 170-172 °C (ether/hexane); R_f_ = 0.69 (ether);^1^H-NMR (CDCl_3_, ppm): δ = 7.78 (m, 2H, H_arom_), 7.55-7.30 (m, 3H, H_arom_), 6.90-6.38 (m, 4H, NH_amid_ + H_arom_), 6.15 (d, 1H, H_α_, 7.8Hz), 5.10 (br s,1H, NH), 3.85 (s, 3H, OCH_3_), 3.80 (s, 3H, OCH_3_), 3.74 (s, 3H, OCH_3_); ^13^C-NMR (CDCl_3_, ppm): δ = 170.5, 167.2 (CO), 153.7, 148.7, 133.4, 132.0, 128.6 (2C), 127.8, 127.4 (2C), 112.9, 103.9, 99.3 (C_6_H_5_ aromatic carbons), 61.3 (-CH-), 55.6, 55.5, 53.2 (OCH_3_); MS (electrospray) *m/z*: 345.2 (M + 1, 17.45%), 224.2 (79.19%), 192.2 (3.36%), 164.3 (11.41%); (Formula: C_18_H_20_N_2_O_5_).

*Methyl 2-benzamido-2-(4-chloro-2-fluorophenylamino)acetate* (**6**): Yield 90%; Solid, m.p. 152-154 °C (ether/hexane); R_f_ = 0.87 (ether);^1^H-NMR (CDCl_3_, ppm): δ = 7.78 (m, 2H, H_arom_), 7.62-7.41 (m, 3H, H_arom_), 7.05-6.85 (m, 4H, NH_amid_ + H_arom_), 6.20 (m, 1H, H_α_), 5.15 (br s, 1H, NH), 3.85 (s, 3H, OCH_3_); ^13^C-NMR (CDCl_3_, ppm): δ = 169.8, 167.3 (CO), 152.6, 132.9, 132.3, 131.5, 128.8 (2C), 127.3 (2C), 124.9, 123.7, 115.7, 114.6 (C_6_H_5_ aromatic carbons), 60.0 (-CH-), 53.5 (OCH_3_); MS (electrospray) *m/z*: 361.1 (6.41%), 359.1 (16.99%), 337.1 (M + 1, 7.05%), 218.2 (32.05%), 216.2 (100%), 122.3 (58.33%); (Formula: C_16_H_14_ClFN_2_O_3_).

*Ethyl 2-benzamido-2-(2-nitrophenylamino)acetate* (**7**): Yield 86%; Solid, m.p. 173-174 °C (ether/hexane); R_f_ = 0.83 (ether);^1^H-NMR (CDCl_3_, ppm): δ = 8.85 (br s, 1H, NH_amid_), 8.25-6.92 (5m, 9H, H_arom_), 6.82 (m, 1H, H_α_), 6.45 (br s, 1H, NH), 4.35 (q, 2H, OCH_2_, 7.1Hz), 1.35 (t, 3H, CH_3_, 7.1Hz); ^13^C-NMR (CDCl_3_, ppm): δ = 168.5, 167.1 (CO), 142.0, 136.6, 133.6, 132.9, 132.4, 128.8 (2C), 127.2 (2C), 126.8, 117.8, 115.1 (C_6_H_5_ aromatic carbons), 63.1 (-CH-); 59.3 (CH_3_CH_2_O); 14.0 (CH_3_CH_2_O); MS (electrospray) *m/z*: 366.2 (26.75%), 344.1 (M + 1, 17.52%), 223.2 (47.77%), 206.2 (57.96%), 105.3 (100%); (Formula: C_17_H_17_N_3_O_5_).

*Ethyl 2-benzamido-2-(naphthalen-2-ylamino)acetate* (**8**): Yield 62%; Solid, m.p. 210-212 °C (ether/hexane); R_f_ = 0.67 (ether);^1^H-NMR (CDCl_3_, ppm): δ = 7.85 (m, 2H, H_arom_), 7.71-7.64 (m, 3H, H_arom_), 7.51-7.25 (m, 6H, H_arom_), 7.07 (m, 2H, NH_amid_ + H_arom_), 6.87 (d, 1H, NH, 8.0Hz), 6.25 (d, 1H, H_α_, 8.0Hz), 4.35 (q, 2H, OCH_2_, 7.1Hz), 1.34 (t, 3H, CH_3_, 7.1Hz); MS (electrospray) *m/z*: 697.3 (2M+1, 4.17%), 349.2 (M + 1, 8.01%), 154.3 (3.21%), 228.1 (100%); (Formula: C_21_H_20_N_2_O_3_).

*Methyl 2-benzamido-2-(benzylamino)acetate* (**9**): Yield 76%; Solid, m.p. 104-106 °C (ether/hexane); 6.97 (m, 1H, NH), 5.56 (d, 1H, H_α_, 7.5Hz), 3.87 (d, 2H, CH_2_, 9.8 Hz), 3.79 (s, 3H, OCH_3_); ^13^C-NMR (CDCl_3_, ppm): δ = 170.8, 167.4 (CO), 139.2, 133.4, 132.1, 128.7 (2C), 128.5 (2C), 128.3 (2C), 127.3, 127.1 (2C) (C_6_H_5_ aromatic carbons), 65.0 (-CH-), 53.0 (OCH_3_), 49.2 (CH_2_); EIMS *m/z*: 298.8 (M^+.^, 100%), 239 (25%), 178 (67%), 105 (31%), 91 (24%), 77 (10%); (Formula: C_17_H_18_N_2_O_3_).

*Methyl 2-benzamido-2-(N,N-dibenzylamino)acetate* (**10**): Yield 84%; Solid, m.p. 130-132 °C (ether/ hexane); R_f_ = 0.82 (ether); ^1^H-NMR (CDCl_3_, ppm): δ = 7.87 (d, 1H, NH_amid_, 7.3 Hz), 7.50 (m, 15H, H_arom_), 5.58 (d, 1H, H_α_, 7.3 Hz), 3.95 (br s, 4H, NCH_2_), 3.85 (s, 3H, OCH_3_); ^13^C-NMR (CDCl_3_, ppm): δ = 170.4, 167.9 (CO), 139.6 (2C), 133.2, 132.2, 129.0 (4C), 128.8 (4C), 128.6 (2C), 128.3 (2C), 127.0 (2C) (C_6_H_5_ aromatic carbons), 66.5 (-CH-); 54.1 (2C) (CH_2_), 52.7 (OCH_3_); EIMS *m/z*: 389.9 (M + 1, 25.20%), 388.9 (M^+.^, 100%), 329.3 (68.50%), 268.3 (93.70%), 196.2 (41.73%), 105.2 (22.05%), 77.3 (55.91%); (Formula: C_24_H_24_N_2_O_3_).

*Methyl 2-benzamido-2-(cyclohexylamino)acetate* (**11**): Yield 92%; Solid, m.p. 182-184 °C (ether/ hexane); R_f_ = 0.6 (ether); ^1^H-NMR (CDCl_3_, ppm): δ = 8.02-7.99 (m, 2H, NH_amid_ + H_arom_), 7.56-7.46 (m, 4H, H_arom_), 5.72 (d, 1H, H_α_, 6.18Hz), 3.87 (s, 3H, OCH_3_), 2.83-0.87 (m, 12H, H_cyc_ + NH); ^13^C-NMR (CDCl_3_, ppm): δ = 170.9, 167.4 (CO), 133.4, 132.0, 128.7 (2C), 127.4 (2C) (C_6_H_5_ aromatic carbons), 63.0 (-CH-), 56.0 (OCH_3_), 52.7 (-CHCH_2_-), 32.5 (2C), 26.3 (2C), 25.2 (CH_2_); EIMS *m/z*: 289.1 (M^+.^, 14.29%), 231.2 (100%), 170.2 (47.62%), 105.4 (88.10%), 77.4 (28.17%); (Formula: C_16_H_22_N_2_O_3_).
